# Effect of Hypoxic Stress and Levels of Mn on the Physiology and Biochemistry of *Phyllostachys praecox*

**DOI:** 10.3390/toxics10060290

**Published:** 2022-05-27

**Authors:** Jiawei Ma, Gul Rukh, Zhengqian Ye, Xiaocui Xie, Zhongqiang Ruan, Dan Liu

**Affiliations:** 1 Key Laboratory of Soil Contamination Bioremediation of Zhejiang Province, Zhejiang A&F University, Hangzhou 311300, China; chaoticant@outlook.com (J.M.); yezhq@zafu.edu.cn (Z.Y.); xxc3043862750@163.com (X.X.); ruanzhognqiang@163.com (Z.R.); 2 The Nurturing Station for the State Key Laboratory of Subtropical Silviculture, Zhejiang A&F University, Hangzhou 311300, China; 3 Department of Chemistry, The Islamia College University Peshawar, Peshawar 25120, Pakistan; gulrukhshafi@gmail.com

**Keywords:** hypoxia stress, Mn^2+^, physiology and biochemistry, *Phyllostachys praecox*

## Abstract

Hypoxic environments have an adverse effect on the growth and development of *P. praecox*, and this is accompanied by the production of reducing substances such as Fe and Mn. In this study, the effect of hypoxic stress and Mn concentrations on leaf chlorophyll contents, root morphology, root activity, element absorption, antioxidant enzymes, and respiratory enzyme system of *P. praecox* were evaluated in a hydroponics environment. The results revealed that application of Mn^2+^ during hypoxic stress enhanced leaf chlorophyll contents and boosted up the indexes of the root system. The root activity of *P. praecox* was reduced with stresses of hypoxia. The treatment of Mn^2+^ initially improved and then decreased the root activity of *P. praecox*, and attained its maximum with application of 300 μmol/L Mn^2+^ compared with control. The indexes of antioxidant enzymes of *P. praecox* were higher than that of 8 mg/L oxygen concentrations except for variable superoxide dismutase (SOD) in the treatment of 300 μmol/L Mn^2+^ with hypoxia stress. The application of Mn had inhibited the absorption of mineral elements in *P. praecox*. The activities of pyruvate decarboxylase (PDC), alcohol dehydrogenase (ADH), and lactic dehydrogenase (LDH) were initially improved and then diminished with hypoxia stress. It is concluded that hypoxia is a key factor affecting the growth and degradation of *P. praecox*, while combining it with the increase of Mn concentration enhances the damage to *Phyllostachys pubescens*. Our research is helpful for the sustainable management and scientific fertilization management of *Phyllostachys praecox*.

## 1. Introduction

Soil aeration is an important aspect of soil quality and soil ecology. Good soil aeration ensures plant health growth and root activity [1,2]. The permeability of soil and supply of oxygen in rhizosphere is affected by soil texture, precipitation, ploughing, and compaction which have influenced the growth and development of plants [3,4]. Adequate oxygen supply is one of the requirement for higher plants to maintain their normal physiological metabolism [5], normal growth, and development processes, which provide energy for plant cells [6,7]. Hypoxic stress is an important factor of abiotic stresses [8,9]. As the endogenous diffusion molecule, oxygen in plants has the signal function of connecting development processes and metabolism activity, which affect plant development pathway [10,11].

Mn is an essential trace element in process of plant growth and development, which is involved in important metabolic reactions in plants [12]. The roots of plants will reduce the redox potential and soil pH value, decrease Mn^4+^ to Mn^2+^, and lead to Mn^2+^ accumulation, which is the main form of plant absorption and utilization. If the plant absorbs too much Mn^2+^, it will inhibit growth, crop yield and quality, and even can endanger the survival of plants [13]. Mn is a cofactor of Mn-SOD, Mn-CAT, pyruvate carboxylase, and phospho enolpyruvate carboxykinase [14]. Therefore, it is essential for cells to absorb Mn. The Mn plays a key role as a positive charge equivalent accumulator in the reaction catalyzed by photosystem II in photosynthesis [15].

Covering Phyllostachys praecox can increase soil temperature in winter, while the growth time of bamboo shoots was advanced by 4 months. During the Spring Festival, which can achieve great economic benefits in the southern region of China, it has realized the poverty alleviation of ten thousand farmers. The typical characteristics of covering treatment for *P. praecox* is the covering of organic matter as a heating layer and heat preservation layer in winter, which isolates the soil from the atmosphere and reduces air convection, in addition to this, fermentation oxygen consumption of organic matter occurs in heating layer at the bottom of cover layer [16,17]. The soil of *P. praecox* is seriously acidified, while Mn of the acid soil increases. This will be the limiting factor for plant growth [18]. It has been reported that the soil oxygen content in the surfaces of covered bamboo is diminished after being covered by bamboo forest. The oxygen content of soil on surface of covered bamboo forest is improved after aeration, which was near to that of an uncovered sample plot [16]. The decomposition rate of organic materials in the later stage of mulching, with rising temperature has enhanced soil respiration which promoted microorganisms and maximum oxygen consumption. The demand for oxygen was increased with the rapid growth of female bamboo [19,20]. It can be inferred that a low oxygen environment was generated, especially in the middle and late stage of coverage, which was accompanied by the production of reducing substances of Fe and Mn [21]. With the continuous management of covering for many years, *P. praecox* during mulching may be in a double stress environment, which limits the sustainable management of *P. praecox* and affects the economic benefits. However, there are few reports on these studies.

To study whether hypoxia and Mn was a key driving factor affecting growth and degradation of *P. praecox*, we studied the root morphology, root activity antioxidant, enzyme index, and anaerobic respiratory enzyme activity of *P. praecox* with different Mn levels and hypoxia stress. This will provide a theoretical basis for exploring the stress resistance mechanism of *P. praecox* under hypoxia stress and different Mn treatment.

## 2. Materials and Methods

### 2.1. Experimental Materials

The tested plants were 1 m tall seedlings of *P. praecox*, collected from *P. praecox* forest in Lin’an, Zhejiang Province. The seedlings were washed with distilled water, three branches were cut, and these were left for pre-culture. All seedlings were cultured with 1/2 Yoshida nutrient solution. Nutrient solution was renewed once every five days. The pH of the nutrient solution was adjusted to 5.8 with 0.1 M NaOH and 0.1 M HCl. Ventilation was maintained for 24 h. The hypoxia treatment was initiated with the growth of new roots. The purity of all chemicals used in the experiment was analytical purity.

### 2.2. Hydroponics Experiment

The seedlings of *P. praecox* with a uniform size were selected for hydroponics experiments. The nutrient solution was set with 2 levels of oxygen concentration and 4 levels of Mn concentration. The total number of treatments was 8 and there were 3 plants in each pot. The nutrient solution was changed repeatedly after every 5 days. The pH value was adjusted to 5.8 with 0.1 M HCl or NaOH on daily basis. The plants were harvested on day 15. The configuration of different Mn treatment concentrations was applied as MnCl_2_ at 0, 10, 300 and 600 μmol/L; the nutrient solution was set with two oxygen concentrations of 2 and 8 mg/L (CK) (Hypoxia treatment: nitrogen was introduced in nutrient solution; The dissolved oxygen controller was used to maintain low oxygen concentration of 2 mg/L; CK: The normal ventilation of ventilation pump had maintained oxygen concentration of 8 mg/L).

### 2.3. Determination of Leaf Chlorophyll Contents (SPAD)

The determination of leaf chlorophyll contents was performed according to Liu’s method [22] using SPAD 502. The relative chlorophyll content was measured at two-thirds of fresh leaves from the leaf margin and three leaves were randomly determined for each plant. The SPAD parameters were measured in vitro in the leaves of plants. Each leaf was repeated for 3 readings.

### 2.4. Determination of Plant Root Morphology

Three root systems with the same condition were selected from each treatment group for the scanning of root morphology. The analysis indexes of root morphology include root length, surface area, average diam, volume, and tips. In this experiment, an Epson expression 10,000xl image scanning system and win-rhizo root analysis software were used for analysis of above indexes [23].

### 2.5. Determination of Plant Root Vitality

The plants with similar growth were selected for root vitality. The roots were washed with distilled water, and the root activity were measured with the TTC method [24]. The fresh roots samples of 0.5 g were homogenized with 10 mL of 0.4% 2,3,5-triphenyltetrazolium chloride (TTC) solution and phosphoric acid buffer, with the root being fully immersed in the solution. The roots were kept in the dark at 37 °C for 1 h, and 2 mL of 1 mol/L sulfuric acid was added to stop the reaction. Then roots were removed from the dark and grounded with ethyl acetate 3–4 mL and a small amount of quartz sand to get 1,3,5-Triphenylformazan (TTF). The red extraction solution was transferred to a test tube. The residue was washed with small amount of ethyl acetate for three times, and transferred to a test tube. The volume was finally adjusted to 10 mL, the color was compared with a spectrophotometer at 485 nm; the OD was read out with a blank as reference; and the standard curve was checked and reduction amount of tetrazolium was calculated.

### 2.6. Determination of Concentrations of Lipid Peroxidation

MDA concentrations were determined according to Peng’s method [25]. The 1 g root samples were grounded with 10 mL 0.1% (*w*/*v*) trichloroacetic acid (TCA) in ice bath conditions. The homogenate was centrifuged at 4000 r/min for 20 min and supernatant was utilized for next chromogenic reaction with thiobarbituric acid (TBA). This test had determined malonaldehyde (MDA) as an end product of lipid peroxidation, which was used for the measurement of lipid peroxidation in samples [26].

### 2.7. Assay of Antioxidant Enzymes

The fresh tissues of roots (about 0.5 g) were homogenized with a mortar and pestle with 5 mL chilled sodium phosphate buffer (50 mM, pH 7.8). The homogenates were centrifuged at 10,000× *g* for 20 min at 4 °C. The supernatant was used for determination of superoxide dismutase (SOD) and peroxidase (POD) activity. Superoxide dismutase activity was determined using the photochemical method as described by a previous report [27]. One unit of SOD activity was defined as the amount of enzyme required to inhibit the reduction rate of NBT by 50%, as measured at 560 nm. The activity of POD was determined using the method as described by a previous study [28]. The 0.1 mL supernatant was used for analysis. The activity was measured by an increase in absorbance at 470 nm due to guaiacol oxidation.

### 2.8. Determination of Proline Concentration

The concentration of proline was determined according to the method of [29] with acid ninhydrin solution. Fresh tissues of root (about 0.5 g) were homogenized with 5 mL 3% sulfosalicylic acid and were extracted in a boiling water bath for 10 min. The 2 mL aliquot of supernatant was transferred to a test tube and 2 mL acetic acid and 2 mL acid ninhydrin were added, and the mixture was boiled for 30 min. After cooling down, the reaction mixture was extracted with 4 mL toluene and mixed thoroughly with shaking. The optical density of upper toluene phase was determined at a wavelength of 520 nm.

### 2.9. Mineral Element Analysis

Roots and leaves were dried at 70 °C for 72 h. The oven-dried plant parts were passed through a 0.1 mm nylon sieve for K, Mg, Cu, Zn, Mn, and Fe analysis. Approximately 0.3 g of roots or leaves was digested in the HNO_3_/HClO_4_ solution and fixed the volume to 50 mL with de-ionized water. The digested solution was washed in 50 mL flasks and volume was made using de-ionized water. The supernatant was analyzed by flame atomic absorption spectrometry (FAAS, PerkinElmer AA800, Los Angeles, CA, USA).

### 2.10. Determination of Anaerobic Respiratory Enzyme Activity

Pyruvate decarboxylase (PDC), lactic dehydrogenase (LDH), and alcohol dehydrogenase(ADH) active enzyme solution were extracted, referring to methods of Mustroph and Albrecht [30]. The 20 mL precooled 50 mmol/L Tris-HCl extract (pH 6.8, containing 5 mmol/L MgCl_2_, 5 mmol/L β-Hydroxy-1-ethanethiol, 15% (*v*/*v*) glycerol, 1 mmol/L EDTA, 1 mmol/L EGTA, and 0.1 mmol/L Phenylmethylsulfonyl fluoride) were added to a root sample of 0.5 g in a precooled mortar. The sample was grounded into homogenate under ice bath, and then the homogenate was transferred to a centrifugal test tube. The centrifugation was conducted at 40 °C and 13,000 r/min for 20 min. The supernatant was collected as crude enzyme solution and enzyme activity was determined. The determination of PDC, ADH, and LDH activity was according to the method of Waters [31], and the determination of protein content was according to the method of Bradford [32]. The calculation of enzyme activity was based on the change of OD value per minute 0.01, which was an enzyme activity unit (U).

### 2.11. Statistical Analysis

Statistical analysis was carried out with statistical package of SPSS (SPSS Inc., Chicago, IL, USA). All values reported were means of at least three independent replicates. Data were tested with a significance level of *p* < 0.05 by a one-way analysis of variance (ANOVA). Graphical work was carried out with Sigma Plot software v.12.5 (Systat Software, San Jose, CA, USA).

## 3. Results

### 3.1. Effect of Hypoxic Stress and Mn on SPAD Values of P. praecox in Hydroponics Environment

**Figure 1:**
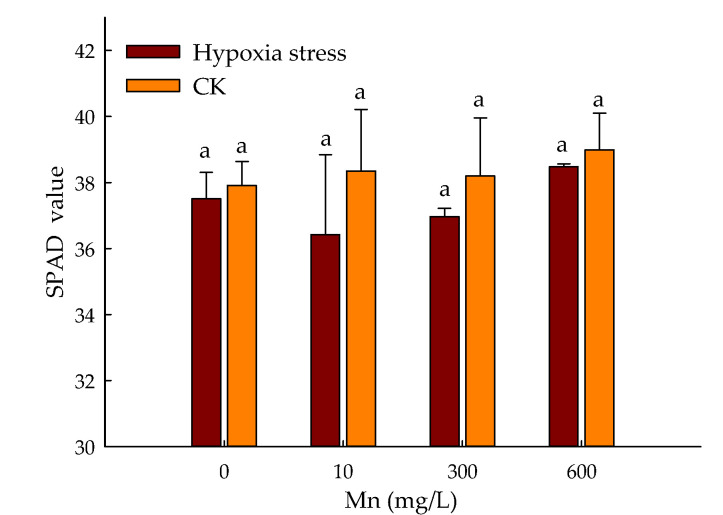
Changes of SPAD values in leaves of *P. praecox* treated with different Mn concentration under hypoxia stress. Data points and error bars represent mean ± S.D. of three replicates (*n* = 3). Different letters indicate significant difference (*p* < 0.05).

The SPAD values in the leaves of *P. praecox* were dwindled due to stresses of hypoxia when compared with control (Figure 1). The SPAD values of *P. praecox* leaves were enhanced by 2.59% at 600 μmol/L Mn^2+^ treatment during hypoxia stress. The Mn had a definite effect on the chlorophyll content of *P. praecox* leaves with hypoxia stress, but no significant variation was observed in SPAD values with CK.

### 3.2. Effect of Hypoxia Stress and Level of Mn on Root Morphology of P. praecox in Hydroponics

The treatments of hypoxic stress revealed variable effects on the length, surface area, average diameter, volume, and tips of roots, except for the root average diameter of *P. praecox* violaceus (Table 1). The length, surface area, volume, and tips of root were consistently improved by 18.32, 23.48, 25.36, and 1.01%, respectively, and attained maximum levels with the treatment of 600 μmol/L Mn^2+^. The root length and root tips were enhanced significantly with hypoxic stress. The root average diameter was initially improved by 15.96% with application of 300 μmol/L Mn^2+^ and then decreased. The root length and average diameter increased, while root surface area, volume, and tips decreased first and then increased in control treatment.

### 3.3. Effect of Hypoxic Stress and Level of Mn on Root Vitality of P. praecox in Hydroponics

**Figure 2:**
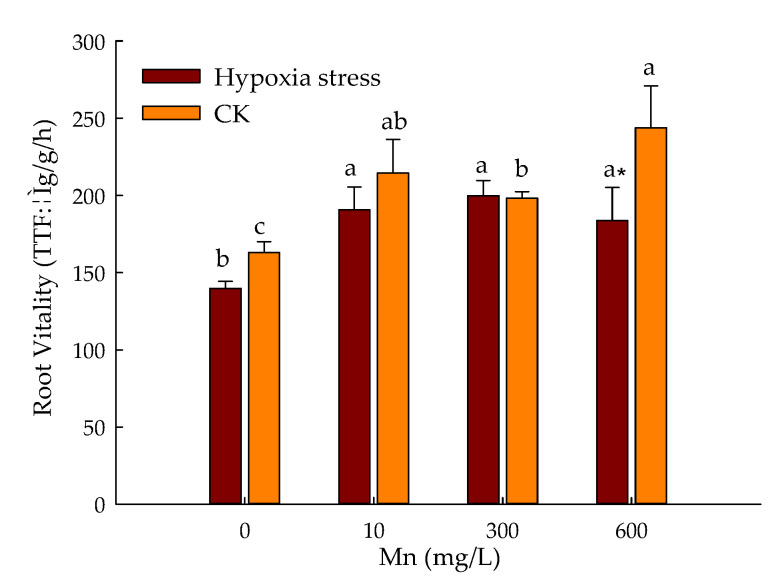
Changes of root activity of *P. praecox* treated with different levels of Mn and hypoxia stress. Data points and error bars represent mean ± S.D. of three replicates (*n* = 3). Different letters indicate significant difference, * indicates the difference between hypoxia stress and CK. (*p* < 0.05).

Figure 2 reveals the root activity of *P. praecox,* which was reduced by 11.1% with the treatment of 10 μmol/L Mn^2+^ with hypoxic stress when compared with control. The root activity of *P. praecox* was initially improved up to 199.79 ± 9.84 μg/g/h with the increase of Mn^2+^ (300 μmol/L) due to hypoxic stress and then decreased, but there was no significant variation. The root activity of *P. praecox* was initially reduced and then enhanced and attained a level of 243.82 ± 27.04 μg/g/h with the application of 600 μmol/L Mn^2+^ in treatment of CK.

### 3.4. Effect of Hypoxic Stress and Levels of Mn on Antioxidation System of P. praecox in Hydroponics Environment

**Figure 3:**
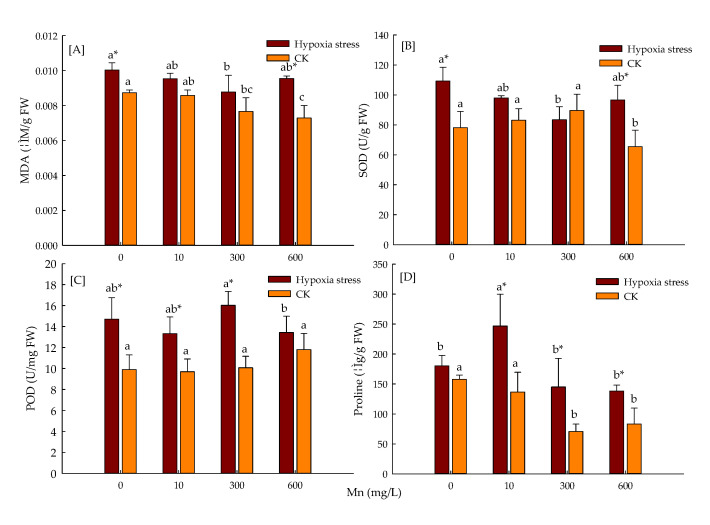
Activity of SOD (**B**) and POD (**C**), and concentrations of MDA (**A**) and proline (**D**) of *P. praecox* during 15 days of hypoxic stress. Data points and error bars represent mean ± S.D. of three replicates (*n* = 3). Different letters indicate significant difference, * indicates the difference between hypoxia stress and CK. (*p* < 0.05).

Figure 3 exhibits the effect of Mn and hypoxic stresses on antioxidant enzyme indexes of *P. praecox*. The antioxidant enzyme indexes were higher than the CK treatment except SOD with 300 μmol/L Mn^2+^ treatment due to hypoxia stresses. The MDA and SOD were reduced with the increase of Mn^2+^. The POD and proline were initially enhanced and then reduced. The MDA, SOD, and proline were dropped by 12.59 and 23.65% respectively, however POD was enhanced by 20.3% with the treatment of hypoxic stress and 300 μmol/L Mn^2+^. The maximum value of increase in proline was 36.99% with application of 10 μmol/L Mn^2+^. The MDA and SOD were increased, while POD and proline decreased with the application of 600 μmol/L Mn^2+^ in treatment of CK. The MDA and proline were decreased significantly by 12.18 and 55.29% respectively with an increase of Mn^2+^ up to 300 μmol/L. The SOD was initially improved and then dropped; however a consistently rising level of POD was observed.

### 3.5. Effect of Hypoxic Stress and Levels of Mn on Mineral Element Absorption of P. praecox in Hydroponics Environment

**Figure 4:**
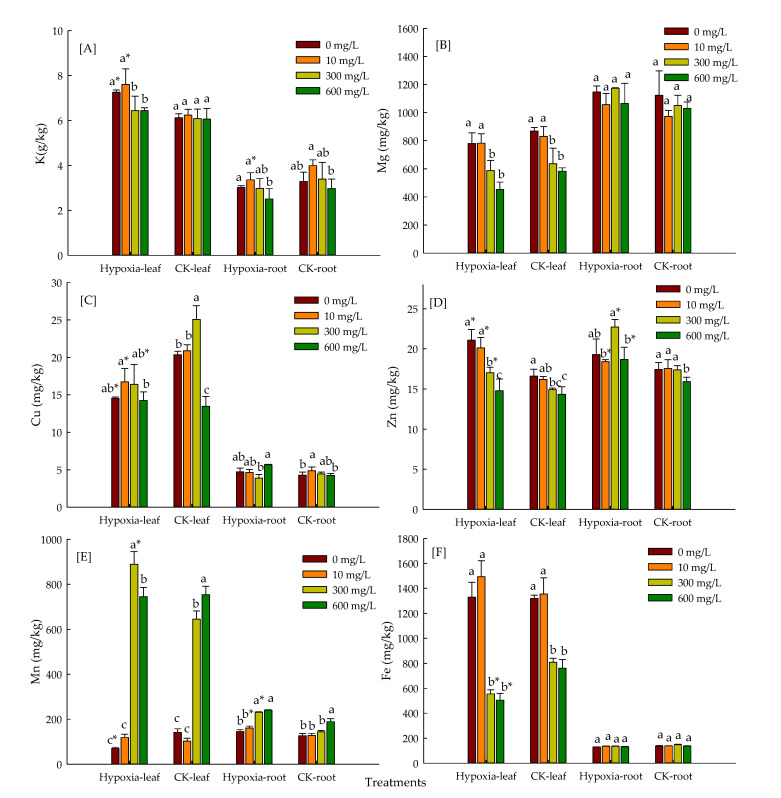
The content of mineral elements K(**A**), Mg(**B**), Cu(**C**), Zn(**D**), Mn(**E**) and Fe(**F**) in *P. praecox* during 15 days of hypoxic stress. Data points and error bars represent mean ± S.D. of three replicates (*n* = 3). Different letters indicate significant difference (*p* < 0.05), * indicates the difference between hypoxia stress and CK.

The hypoxic stress resulted in the reduced accumulation of Cu in leaves (Figure 4). The leaves and CK roots were initially raised and then exhibited a decreasing trend with an increase of Mn^2+^. The hypoxic roots exhibited initially a declining trend and then increased. The absorption and accumulation of K and Zn by *P. praecox* was reduced significantly. The K and Zn content in the root was firstly improved and then decreased with increase of Mn^2+^. The Mn content in *P. praecox* was firstly enhanced and then decreased with increase of Mn^2+^ under hypoxic stress. The maximum value of increase in Mn content was 13.63 times higher than the control in treatment of 300 μmol/L Mn^2+^. There was a maximum value of increase 65.13, 433.33, and 49.23%, respectively with 600 μmol/L Mn^2+^ in CK-leaf and CK-root. There was no significant change in Mg and Fe content between 0 and 10 μmol/L Mn^2+^. The contents of Mg and Fe were reduced significantly in leaf due to enhancing Mn, while there was no significant change in roots.

### 3.6. Effect of Hypoxic Stress and Levels of Mn on Anaerobic Respiratory Enzyme Activity of P. praecox in Hydroponics Environment

**Figure 5:**
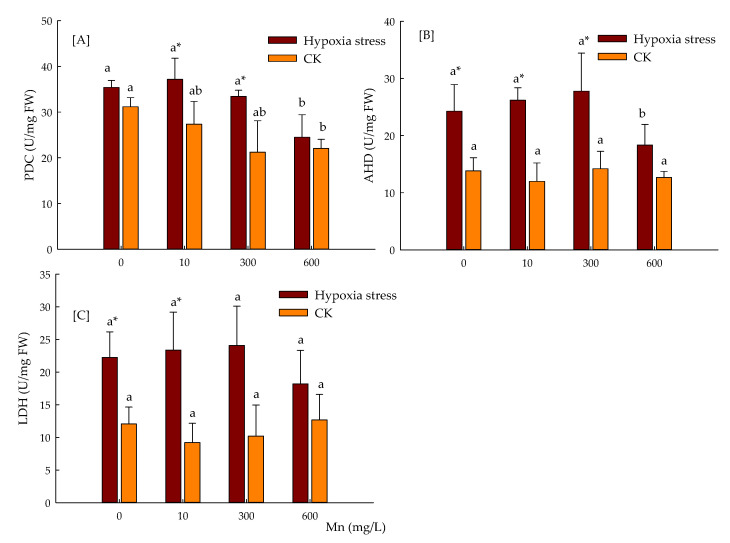
Activity of PDC (**A**), ADH (**B**), and LDH (**C**) of *P. praecox* during 15 days of hypoxia stress. Data points and error bars represent mean ± S.D. of three replicates (*n* = 3). Different letters indicate a significant difference, * indicates the difference between hypoxia stress and CK. (*p* < 0.05).

Figure 5 reveals the higher activity of PDC, LDH, and ADH than CK in hypoxia stress. The PDC, LDH, and ADH activity was initially enhanced and then fell with increases of Mn^2+^. The maximum value of improvement in PDC activity was 5.09% with application of 10 μmol/L Mn^2+^; LDH and ADH activity with an application of 300 μmol/L Mn^2+^ was 14.42 and 8.24%, respectively. The minimum value of decrease in enzyme activities was observed under 600 μmol/L Mn^2+^ stress, which were reduced by 30.74, 24.47, and 18.10%, respectively, compared to without Mn in CK conditions. The PDC activity declined with the increase of Mn^2+^. The LDH and ADH activity were not changed significantly.

## 4. Discussion

Chlorophyll is an essential pigment for photosynthesis of plants [33]. Hypoxia stress affects chlorophyll biosynthesis and photosystem II (PSII) by reducing chlorophyll content [34]. The SPAD value is an indicator of the response of plant photosynthesis to variable environments [35]. In this study, the photosynthetic mechanism of *P. praecox* was destroyed and photosynthetic rate was reduced significantly by hypoxic stress, as being illustrated by previous studies [8]. However, the addition of Mn has effectively protected the photosynthetic mechanism from damage, and then adapted it to an hypoxic environment [36].

The root system is very crucial for the absorption and utilization of water and nutrients by plants. The length, surface area, average diameter, volume, and tips of roots reflect the degree of root development [37]. The high rhizosphere had dissolved oxygen content (8.0–9.0 mg/L), promoting root growth and development in the split-root system [38]. In this study, the condition of hypoxia had significantly decreased indexes of plants than ventilation treatment. This is due to inhibition of aerobic respiration of plant cells with an environment of hypoxia, which produced an underdeveloped root system. Coarser and shorter rice roots grown in an environment of poor ventilation was reported by previous study [39]. This is probably due to the adaptive mechanism of root systems to hypoxia [40]. The addition of Mn can obviously improve root growth, promoting the elongation, volume, surface area, and number of root tips, but reducing root diameter due to the stresses of hypoxia. The application of Mn could promote root growth via enhancing the absorption of water and nutrients. The root system is an important organ for plant absorption and metabolism. Growth and development of roots directly affects the growth of shoots and leaves. The root activity is a comprehensive index reflecting root absorption function [41]. In this study, the root activity of *P. praecox* was reduced due to stresses of hypoxia, which indicated its damage to the root system. Higher root activity is beneficial to maintain function of the root system and plant growth, and to enhance plant resistance during condition of adversity [42]. In this study, 300 μmol/L Mn^2+^ treatment increased the root activity of plants, and but decreased root activity with high concentrations. This may be due to the improved resistance of *P. praecox* to hypoxic stress with application of 300 μmol/L Mn^2+^. The Mn^2+^ concentration ranged between 10 and 200 M, which had an improved variability of the root cell, but reversely, the high level of Mn^2+^ aggravated the existing damage to seedlings of tomato [43].

MDA is the final product of membrane lipid peroxidation, which usually reflects the degree of membrane lipid peroxidation and the adaptability of plants to stress [44]. The results revealed that MDA contents in plant was higher than CK by hypoxia stress [43]. The addition of Mn had reduced MDA content. Therefore, improvement of MDA and degree of lipid peroxidation of cell membrane can be inhibited by the application of Mn with hypoxic stress, which is of positive significance for seedling growth [13].

The SOD and POD activities are important protective enzymes defense system for protection of cells from oxidative damage [12]. This study reveals that antioxidant enzymes were enhanced with an increase of Mn. The POD activity was initially increased and then declined, while the trend of SOD was opposite, which showed that 300 μmol/L Mn^2+^ increased POD activity and protected it from oxidative damage caused by hypoxia stress. Yet 600 μmol/L Mn^2+^ treatment decreased POD activity, which may be due to poisoning plant cells with high concentrations of Mn [13]. The treatment of 300 μmol/L Mn^2+^ reduced SOD activity, which has protected plant cells from oxidative damage.

Proline will accumulate in plants in adverse environments. The content of proline in plants reflects the tolerance of plants to adversity in some extent [45]. It has been reported that proline was accumulated in plant cells due to hypoxia conditions, while the content of proline was lower due to treatment of aeration [46]. In this study, hypoxia has enhanced accumulation of proline in plants, while high levels of Mn have reduced the content of proline, indicating that increases of Mn protect plant cells from oxidative damage.

Mineral elements play an important role in the material composition and metabolism of plants. Especially, Mn stress alters absorption and distribution of mineral elements in plants [43]. The results showed significant increases in the Mn content of roots and leaves of *P. praecox* with addition of Mn, while content of Mn initially raised and then reduced in hypoxia stress. The high concentration of Mn inhibited the absorption of bamboo under low oxygen stress. The Mn excess inhibits absorption of other essential elements due to the similarity of ion size or binding strength in ligands [47]. In this study, under 600 mn stress, the absorption of Cu, Zn, Mg, and Fe was inhibited. Because Mg and Fe were important components of chlorophyll, the chloroplast structure was destroyed and photosynthesis was affected [48].

Hypoxic stress can enhance the enzyme activity of anaerobic respiration in plants, promoting lactic acid and ethanol fermentation, and the reoxidation process of NADH, as well as cell energy charge, but reducing the acidification of root cells, therefore enhancing the resistance of plants to hypoxia stress [49]. Studies have shown that insufficient oxygen temporarily converts pyruvate into lactic acid, which reduces the cytoplasmic pH, activates POC to produce acetaldehyde, and can be converted into ethanol with the action of ADH. Ethanol can easily diffuse to the external environment through the cell membrane lipid bilayer, reducing the accumulation of acetaldehyde in plants [50,51]. In this study, due to hypoxia stress, the activities of PDC, ADH, and LDH were initially increased and then dwindled. The raise of PDC, ADH, and LDH activities, on the one hand, converts acetaldehyde into ethanol to reduce toxicity of acetaldehyde to cells; on the other hand, it reduces the production of lactic acid and avoids excessive acidification of cells.

## 5. Conclusions

Our work revealed that the SPAD value was enhanced with Mn^2+^ during hypoxia stress. The length, surface area, volume, and tips of *P. praecox* root were always lower than CK except for root average diameter due to hypoxia stress. The addition of Mn^2+^ had boosted up indexes of root system. The root activity of *P. praecox* was reduced with hypoxia stress. The Mn^2+^ treatment initially enhanced the root activity of *P. praecox* and then decreased it, and reached its maximum with the application of 300 μmol/L Mn^2+^ compared with control; The indexes of antioxidant enzymes of *P. praecox* were higher than CK, except for the variation of SOD with the application of 300 μmol/L Mn^2+^ during the stresses of hypoxia. The application of Mn inhibited the absorption of mineral elements by *P. praecox*. The activities of PDC, ADH, and LDH were initially improved and then decreased during hypoxia stress. This study showed the physiological response mechanism of *Phyllostachys praecox* to environmental factors under double stress.

Hypoxia is a key factor to affect degradation, but manganese enhances the damage to *P. praecox*. This finding is helpful for optimal fertilization management and sustainable production in the bamboo forest.

## Figures and Tables

**Table 1:** Root morphology of *P. praecox* as affected by different Mn concentration during hypoxia stress.

	Mn (μmol/L)	Length (cm)	Surf Area (cm^2^)	Avg Diam (mm)	Root Volume (cm^3^)	Root Tips
Hypoxia	0	37.76 ± 1.92 ab	8.74 ± 0.39 b	0.75 ± 0.01 b	0.21 ± 0.08 a	99.00 ± 39.60 a
	10	34.51 ± 1.49 b	9.15 ± 0.11 a	0.80 ± 0.11 a	0.23 ± 0.04 a	67.50 ± 20.51 b
	300	38.19 ± 5.24 ab	10.43 ± 1.85 a	0.87 ± 0.04 a	0.23 ± 0.05 a	80.00 ± 9.90 ab
	600	44.68 ± 4.57 a	10.79 ± 2.72 a	0.76 ± 0.11 ab	0.26 ± 0.02 a	100.00 ± 1.41 a
CK	0	42.85 ± 2.12 a	12.71 ± 0.71 b	0.86 ± 0.05 ab	0.28 ± 0.01 b	106.00 ± 14.85
	10	46.53 ± 3.88 a	14.80 ± 2.25 ab	0.66 ± 0.01 b	0.29 ± 0.06 ab	111.00 ± 22.63 a
	300	47.64 ± 0.96 a	12.33 ± 0.47 b	0.86 ± 0.05 ab	0.27 ± 0.03 b	105.00 ± 13.44 a
	600	51.29 ± 7.42 a	17.92 ± 0.47 a	0.91 ± 0.16 a	0.35 ± 0.03 a	124.50 ± 16.26 a

Note: Different letters indicate significant difference between different treatments in the same period (*p* < 0.05).

## Data Availability

The data presented in this study are available on request from the corresponding author.

## Abbreviations

TCA	Trichloroacetic acid
TBA	Thiobarbituric acid
MDA	Malonaldehyde
SOD	Superoxide dismutase
POD	Peroxidase
PDC	Pyruvate decarboxylase
LDH	Lactic dehydrogenase
ADH	Alcohol dehydrogenase
